# Amino Acids as Targets for Immunotherapy against Solid Tumors

**DOI:** 10.1007/s12013-026-02050-y

**Published:** 2026-03-26

**Authors:** Karine Vitória Viana da Costa, Gabriel Antero Passos, Rodrigo Jardim Soares Botelho, Marina Sobreira Vieira Ruza, Geovane Dias-Lopes, Rafael Cardoso Maciel Costa Silva

**Affiliations:** Faculty of Medical Sciences, State University of Rio de Janeiro, Cabo Frio, Brazil

**Keywords:** Cellular metabolism, Metabolites, Antitumor immunity, Receptors, Immune cells, Tumors cells

## Abstract

The metabolism of tumor cells exerts a great influence on the tumor microenvironment (TME) of solid tumors and antitumor immune responses. Targeting molecules from the TME is an exciting strategy to overcome the ability of solid tumors to escape immunity and immunotherapies. In this sense, this manuscript delves into the complex and sometimes antagonizing role of amino acids in the TME, regulating both tumor cells and immune cells’ biology. Understanding the specific effects of different amino acids in distinct tumors can direct compelling strategies to improve antitumor therapies, especially immunotherapies.

## Introduction

Amino acids are the building blocks for both structural proteins and enzymes that comprise all cells. Some can be synthesized by the human body (non-essential ones), while others need to be obtained through nutrients (i.e., histidine, methionine, threonine, valine, isoleucine, phenylalanine, tryptophan, leucine, and lysine) [1]. The non-essential amino acids are alanine, arginine, asparagine, glutamic acid, aspartic acid, glutamine, cysteine, glycine, serine, tyrosine, and proline [1–3]. Interestingly, some of these nonessential amino acids, like arginine, can also be described as conditionally essential, since in some situations, the ability of the body (cells) to endogenously synthesize them is not enough for its metabolic requirements. Furthermore, amino acids exert a great influence on the cell metabolism, both as carbon and nitrogen sources [3, 4], participating in many distinct metabolic pathways, e.g., the biosynthesis of nucleotides and antioxidant molecules. In addition, it is important to underscore that distinct amino acids can be degraded into distinct metabolites, which exert different functions. In this sense, the amino acids that can be degraded into ketone bodies are known as ketogenic amino acids (leucine and lysine), while those that can be converted to glucose are called glucogenic amino acids (alanine, cysteine, glycine, serine, threonine, valine, histidine, arginine, and methionine) [3]. Tyrosine, isoleucine, phenylalanine, and tryptophan are both glucogenic and ketogenic amino acids [3]. For their obvious importance to cellular homeostasis, including immune cells responses (which are activated by disturbances in homeostasis) [5], amino acids have been used as supplements to improve several medical conditions, for example, burn patients [6]. Besides that, different amino acids in the TME, through their ability to influence cellular biology, have been described to impact both tumor and immune cells functions in solid tumors through many and sometimes unique mechanisms, discussed next [7–9]. Several studies have elegantly reviewed the role of amino acids in tumor biology and immune responses [8, 10–14]. Here, we will focus on the dual effects of amino acids in both tumor and immune cells, highlighting their dichotomous functions depending on the tumor type and infiltrated immune cells. As the metabolic environment surrounding a primary tumor can significantly influence metastasis, the components of the TME are fundamental for the prognosis. For example, in a breast cancer mouse model, limiting asparagine levels effectively decreased metastatic spread without altering the growth of the primary tumor itself. Conversely, an increase in asparagine availability accelerated metastatic progression [5].

Tumor cells and immune cells share metabolic pathways, receptors, and transporters of amino acids, meaning that the final role of certain amino acids in the TME will depend on the type of tumor cells and/or infiltrated immune cells in specific solid tumors [15, 16]. Both the local environment where the tumor cells developed and the type of mutations within these cells will affect their metabolism, also impacting the specific metabolites in the TME and the recruitment of immune cells [15, 16]. In addition, dietary interventions can reshape nutrient availability within the TME, potentially offering a promising strategy to suppress tumor growth [17]. However, this approach requires a deeper understanding of the role of amino acids in distinct tumors. Finally, since the TME in solid tumors exerts great influence on the outcome after immunotherapy, different therapeutic strategies can be used in combination to improve the success of antitumor immunity. Here, it will be discussed how distinct amino acids and linked metabolites (nitro derivatives) can impact different tumors and antitumor immune responses.

### The Impact of Amino Acids in Tumor Biology: General Mechanisms

Since amino acids are critical for the metabolism of both tumor cells and immune cells, tumors can escape immune responses by outcompeting immune cells for the acquisition of amino acids. This way, tumor cells impair immune cell function through metabolic starvation, and, conversely, immune cells can restrain tumor growth or foster tumor elimination by outcompeting tumor cells for amino acids or simply degrading certain amino acids in the TME [18]. However, nutrient starvation can be overcome by adaptive cellular responses, mediated, for example, by General Control Nonderepressible 2 (GCN2), which might actually promote antitumor immunity by specific immune cells or lead to immune escape of tumor cells expressing, for example, PD-L1, as discussed later [19]. Besides that, the induction of mammalian target of rapamycin (mTOR) by certain amino acids (leucine, arginine, lysine, glutamine, methionine, and tryptophan) [20–24] can paradoxically influence immune and tumor cells depending on the TME and infiltrated immune cells [25]. mTOR-mediated signaling promotes cellular proliferation and anabolism, increasing the uptake of carbon and nitrogen sources, like sugars through glucose transporters and amino acids (in a positive feedback way) by the expression of Slc transporters, induced by Activating Transcription Factor 4 (ATF4). Furthermore, activated mTOR can affect other cellular processes involved in the biology of tumor cells and immune cells. For instance, mTOR restrains the activation of AMPK, negatively affecting the macroautophagic process [26]. In this sense, amino acids, through the activation of mTOR, can induce tumor cells’ proliferation, but restrain their ability to adapt to distinct stresses, including hypoxic and nutritional ones [27], after the induction of macroautophagy (from now on, autophagy). On the other hand, the autophagic process can, through the enzymatic degradation of proteins (inside autolysosomes), increase the levels of intracellular amino acids, which are directed to the synthesis of other essential peptides and proteins for cellular adaptation, in a negative feedback mechanism. In this sense, blocking both autophagy and amino acid acquisition in tumor cells can have synergistic antitumor effects [28–30]. However, autophagy, as a catabolic reaction, must be finely regulated, since it can promote cell death if induced excessively, and/or restrain cellular proliferation. It is important to note that the role of autophagy as an inhibitor of cellular proliferation is controversial since inhibitors of autophagy are usually associated with the restriction of tumor cells proliferation, making it difficult to establish a causal role, as it may simply be the consequence of cellular stress (that also causes autophagy) [31]. Thus, in tumor cell biology, autophagy can yield paradoxical results. Similarly, in immune cells, autophagy and mTOR exert paradoxical effects regarding inflammatory or anti-inflammatory responses. For example, although mTOR activation is critical for lysosome tubulation and the surface expression of MHC-II in myeloid cells [32], also promoting effector T cells proliferation and functions, it can negatively impact antigen presentation by APCs after the inhibition of autophagy [33]. Autophagy can promote immune T cells’ memory, antimicrobial activity of immune cells, antigen presentation, and endosomal TLR7 activation [34, 35]; but, in contrast, it can restrain intracellular PRRs signaling (RLRs, NLRP3) and cytokine secretion [34]. The influence of autophagy in both tumor and immune cells will be discussed later under the prism of each amino acid.

Distinct amino acids, like methionine, serine, and alanine, are also critical for the one-carbon metabolism, which supports methyl reactions (named methylation) in DNA and proteins [27]. Therefore, these amino acids can impact the function (signaling or structural) of important proteins involved in tumor and immune cells biology, including histones, leading to epigenetic changes. Additionally, amino acids (methionine, cysteine, arginine, glutamine) can also support the synthesis of antioxidant molecules, like glutathione, which is fundamental for tumor and immune cells adaptation to oxidative stress, and survival, but also affects ROS-dependent signaling, for example.

In these contexts, several studies discussed here demonstrate the paradoxical roles of certain amino acids for antitumor immunity and tumor elimination. Understanding the mechanisms by which amino acids exert their effects on immune or tumor cells can lead to therapeutic advances, targeting enzymes (from the folate cycle, for example), receptors, transporters, molecular partners, or transcription factors involved in these effects. It is also important to bear in mind that distinct amino acids can promote redundant metabolic pathways, meaning that the deprivation of a single one might be offset by the presence of others in the TME [36, 37]. This reinforces that the specific metabolites present in the TME will influence each other’s effects in tumor and immune cells biology. This manuscript aims to underscore that distinct, and sometimes antagonistic, therapeutic approaches should be used for different tumors when focusing on amino acid metabolism, i.e., supplementation, modulation of enzymes involved in metabolism, and/or cellular transporters.

### The Role of Methionine, Cysteine, and Lysine in Tumor Biology

Methionine is an essential amino acid that, along with metabolites from its degradation, e.g., 5-methylthioadenosine (MTA) and S-adenosylmethionine (SAM), is critical for one-carbon metabolism (which generates methyl donors) and for glutathione, purine, pyrimidine, and polyamine synthesis [38]. In this sense, as anticipated, methionine (and also other amino acids discussed later) is crucial for the transfer of single-carbon units to proteins and nucleic acids (dependent on the integration of folate and methionine cycles), fostering proteins’ post-translational modifications (PTMs) and epigenetic changes. Methionine also fosters antioxidant responses (through glutathione generation) in cells. Therefore, methionine, its byproducts, and the transporters and enzymes responsible for their acquisition and generation/recycling exert great influence on both tumor and immune cells. The epigenetic changes associated with methionine metabolism can be the result of both DNA methylation and/or histone methylation, influencing both proliferation and immune escape of tumor cells [39]. In addition, the epigenetic landscape affects the biology of immune cells differently: increasing inflammatory (antitumor) responses, or inhibiting Teff’s function, while promoting suppressor immune cells [40, 41]. Modulators targeting enzymes involved in the methylation process, like PRMTs, have been used as an adjuvant therapeutic strategy in mouse models of sarcoma and breast tumors, improving antitumor immunity and restraining tumor cells proliferation [42–48].

In relation to immune cells, methionine one-carbon metabolism has been linked to CD8 + T cells exhaustion through DNA methylation [49], affecting chromatin accessibility and decreasing the expression of IRF4 (Interferon regulatory factor 4), TCF7 (Transcription Factor 7), RUNX3 (Runt-related transcription factor 3) and CD28 (cluster of differentiation 28), important genes for T cells activation. Simultaneously, it also increases the expression of PDCD1 (Programmed Cell Death 1), which is involved in T cell receptor (TCR) silencing. In this sense, the specific inhibition of enzymes involved in the methionine cycle can be an interesting therapeutic alternative to promote CD8 + T cells activity. Thus, the deletion of the enzyme methionine adenosyltransferase 2 A, crucial for SAM (a methyl donor) synthesis from methionine, was associated with antitumor immunity mediated by T cells in a mouse model of hepatocellular carcinoma [50]. In contrast, at early stages of T cells activation, methionine is critical for the methylation of a calcium-activated potassium transporter (KCa3.1), negatively regulating its function and impairing both excessive activation of NFAT1 and early exhaustion [51]. In this sense, the kinetics and intensity of the signals downstream TCRs are intrinsically linked, meaning that an optimal (neither a very weak nor too strong) stimulus is pivotal for T cells activity, hampering anergy or exhaustion [52]. Methionine is also a critical amino acid for T cells anabolic reactions. Supporting the important role of methionine for the metabolism and function of T cells and NK cells [53, 54], its deprivation from the TME (due to increased acquisition by melanoma cells), contributes to the restriction of T cells activity [54]. Thus, the inhibition of the methionine transporter L-type amino acid transporter 4 (LAT4- or Solute carrier protein 43a2-Slc43a2) in tumor cells, or methionine supplementation, along with the association with Stimulator of Interferon Genes (STING) activators, promotes tumor elimination in a melanoma mouse model [54]. In this setting, STING activators are important to circumvent the anti-inflammatory effects of methionine, related to its ability to indirectly inhibit the STING pathway and subsequent type I interferons (IFN-I) secretion. Methionine restrains IFN-I secretion downstream of cGAS, promoting cGAS (cyclic GMP-AMP synthase) methylation [55]. Methionine supplementation was also critical for epigenetic modifications in CD8 + T cells, STAT5 expression, T cells survival, and cytokines release [55]. Intriguingly, methionine dietary restriction can impair colorectal tumorigenesis in mouse models and immune escape by murine melanoma cells, leading to enhanced cGAS activation, release of IFN-I, increased antigen presentation (in the context of MHC-I), and antitumor immunity. Thus, both methionine restriction and supplementation can be associated with improved antitumor immunity, depending on the TME, i.e., how methionine is metabolized by T cells (for proteins or DNA methylation) and tumor cells, and the presence of STING activators (including metabolites, like fumarate [56]). In macrophages, methionine stimulus promotes M1 polarization in vitro [57], which can release toxic mediators, like reactive oxygen species (ROS), and foster tumor elimination. Nevertheless, the role of methionine on macrophages’ function also seems to be context-dependent, since methionine from apoptotic cells promotes Dual specificity protein phosphatase 4 (Dusp4) methylation and repression, leading to the resolution of inflammatory responses [58]. In the context of tumors, resolution might protect tumor tissue from injury, indirectly promoting tumor growth.

Methionine is also crucial to support tumor cells’ metabolism [11], and its depletion can be associated with Receptor-interacting serine/threonine-protein kinase 1 (RIPK1) activation (due to the loss of methylation of RIPK1 in a specific arginine residue) and subsequent cell death and inflammatory responses [59]. As reviewed by others, inflammation possesses a dichotomous role in tumor biology [60]. Furthermore, the use of the anti-ischemic drug ranolazine promotes polyamine synthesis from SAM (originating from methionine), which reduces the availability of SAM for methylation reactions. This leads to the accumulation of 5’-methylthioadenosine (a natural inhibitor of methyl transferases) in melanoma cells, and an altered epigenetic landscape that reduces cellular proliferation. It also inhibits fatty acid oxidation, reducing acetyl-CoA generation, and is associated with increased type I IFN (IFN-I) signaling, antigen presentation, and programmed death-ligand 1 (PD-L1) expression (which is a target for anti-PD-L1 therapy), improving immunotherapy responses [38, 61]. Annexin A1 expression, mediated by methionine metabolism, can also promote escape mechanisms from immune responses in different models, including glioma cells [62]. Annexin A1 expression in glioma promotes an immunosuppressive phenotype in macrophages (M2), leading to a permissive TME [62]. Thus, the inhibition of enzymes involved in methionine metabolism is an interesting therapeutic opportunity, at least for some tumors, like osteosarcomas and gliomas [62, 63] (Table 1).

**Table 1:** The dual role of methionine, cysteine, and lysine in tumor biology (and immune responses)

Amino acids	Protumor effects	Antitumor effects	Therapeutic opportunities
**Methionine**	Contributes to T cells exhaustion [49]; SAM synthesis, in the presence of methionine, restrains the antitumor activity of T cells [50]; inhibits RIPK1-mediated tumor cell death [59]; induces the expression of annexin A1 and PD-L1 in tumor cells, hampering antitumor immunity [62]; supports tumor cells’ metabolism, especially tumor-initiating cells [259].	At a certain level, it is important to support T cells (especially Th17 cells [260]) and NK cells’ metabolism and the fine-tuning of TCR signaling, preventing excessive activation of T cells and exhaustion [51, 52]; stimulates M1 polarization of macrophages, which possess antitumor activity [57].	Specific delivery of one-carbon metabolism inhibitors to T cells and tumor cells might simultaneously promote tumor cells death and antitumor immunity. Methionine supplementation to T cells improves their antitumor ability, especially if combined with STING activators that enhance the activity of myeloid cells, restrained by methionine-mediated inhibition of cGAS.
**Cysteine**	Supports glutathione synthesis and resistance to oxidative stress, impairing tumor (breast and prostate) cell death [67]; cystine supports antioxidant responses, inhibiting ferroptosis of tumor cells [66].	Supports antioxidant responses in T cells, promoting their survival, proliferation, and (colon cancer and melanoma mouse models) antitumor immunity [68, 69].	Specific delivery of ROS-inducing drugs or glutathione inhibitors to tumor cells can lead to increased cell death.
**Lysine**	Induces epigenetic changes (through histone crotonylation) in CD8 + T cells and DCs, restricting their functions [70]; protects glioblastoma cells from arginine-derived nitric oxide and lysosomal stress [74].	It is critical for T cells’ metabolism and antitumor immunity against hepatocellular carcinoma (mouse model) [72]; its catabolism leads to acetoacetate generation, and subsequently autophagy induction and cell senescence, impairing breast cancer cells’ proliferation [73].	Specific delivery of pharmaceuticals that restrain histone crotonylation can lead to enhanced antitumor immunity.

Methionine also supplies the sulfur atom required for cysteine synthesis. The enzyme nitrogen fixation 1 homolog (NFS1- or cysteine desulfurase) uses cysteine-derived sulfur for iron–sulfur cluster biosynthesis. In lung adenocarcinoma, NFS1 suppression triggers ferroptosis in vitro, resulting in reduced tumor growth [64]. Blocking cystine (an oxidized dimer of cysteine) uptake in tumor cells, including fibrosarcoma and melanoma cell lines, enhances lipid peroxidation and induces ferroptosis [65], while the overexpression of cystine/glutamate antiporter (Slc7a11) promotes tumor growth [66]. Moreover, intracellular glutathione produced from cysteine (and methionine) is a key regulator of oxidative stress. Thus, treatment with the cyst(e)inase enzyme, which depletes cysteine, leads to glutathione loss and increased ROS, ultimately causing cell cycle arrest and tumor cell death in breast and prostate cancer xenografts in mice [67]. On the other hand, both cystine and cysteine are important for T cells’ survival and differentiation under oxidative stress, (cystine) promoting antitumor immunity in colon and melanoma mouse models [68, 69]. These results reinforce the dual role of cysteine in tumor biology /immune responses (Table 1).

**Fig. 1:**
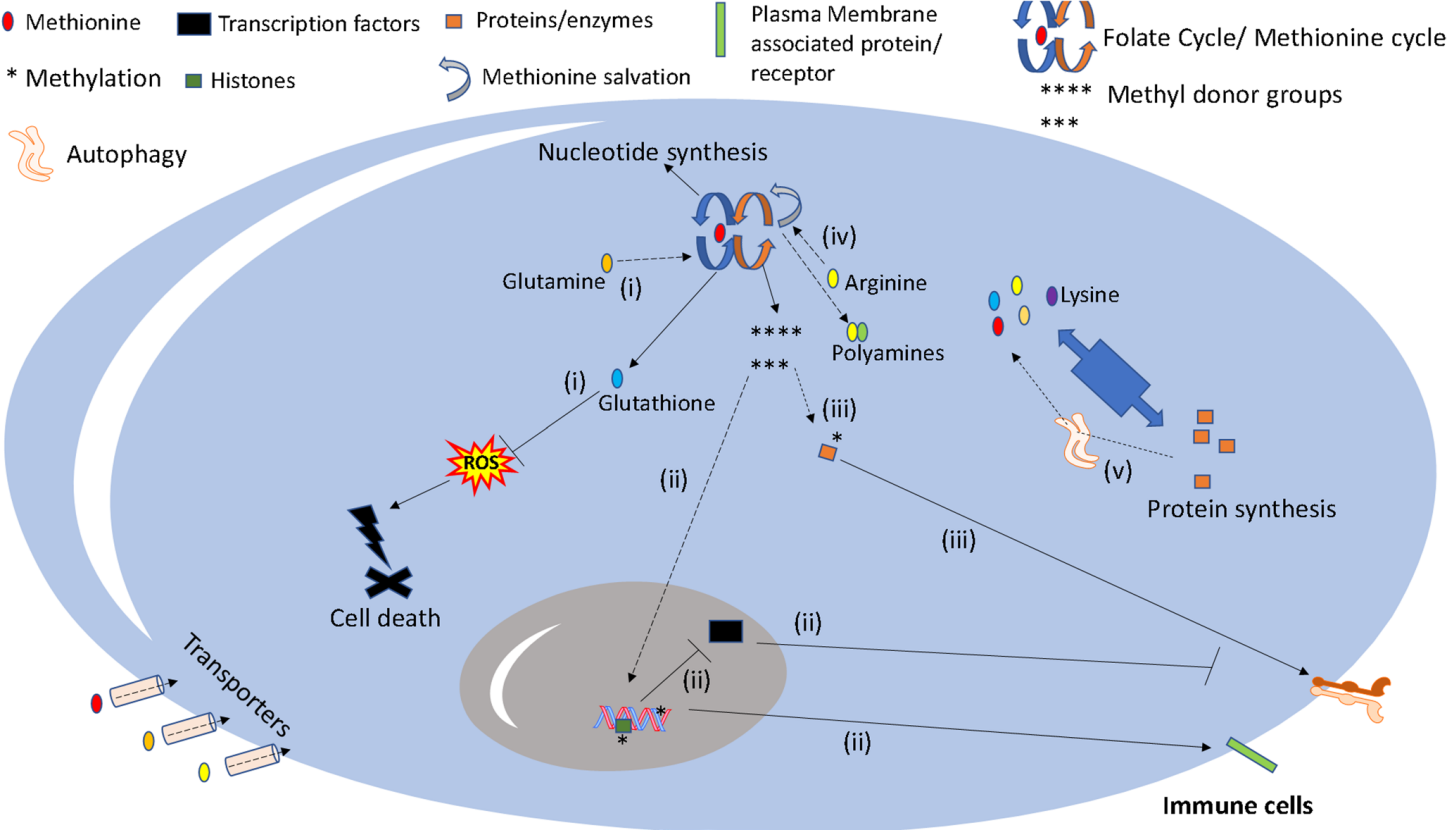
Main mechanisms by which amino acids, focusing on methionine, influence the biology of immune cells. Distinct amino acids can support immune cells’ function or restrain it. (i) Glutamine, serine glycine, cysteine and methionine foster glutathione synthesis, which restrain oxidative stress and cell death, especially ferroptosis of immune cells [260], also promoting M1 polarization of macrophages and antitumor immunity [266]; (ii) The methionine cycle, which is influenced by several other amino acids (e.g., arginine and glutamine) [267], is critical for the generation of SAM and other methyl donor groups that sustain PTMs of proteins and DNA methylation. Through these, methionine restrains CD8 + T cells’ function by promoting PD-1 and hampering CD28, TCF7, and IRF4 expressions [49]. In contrast, methionine-mediated histone methylation (but not DNA methylation) promotes STAT5 expression and CD8 + T cells’ activity against murine melanoma [55]. Regulating the enzymes involved in histone (histone methyltransferases) or DNA methylation (DNA methyltransferases) can promote or impair CD8 + T cells’ function, respectively. Lysine also promotes crotonylation, a form of PTMs, in histones, leading to impaired IFN-I receptor-mediated signaling and reduced CD8 + T cells and DCs activities [70]; (iii) Methionine supports KCa3.1 methylation, which is critical for optimal (early) activation of T cells after TCR signaling [51]. Methionine, from apoptotic bodies, promotes Dusp4 methylation, repressing its enzymatic activity, and promoting macrophage-mediated resolution of inflammatory responses [58]; (iv) Arginine fuels the methionine salvage pathway, which can lead to the synthesis of polyamines. In this context, arginine consumption by TAMs promotes polyamine synthesis and epigenetic alterations associated with immune suppression (PD-L1 expression, IL-10, and TGFβ release) in a mouse model of breast cancer [88]; (v) Amino acids are the building blocks for protein synthesis, also contributing to the synthesis of nucleotides. Therefore, they are critical for proliferating cells, like activated T cells, promoting mTOR activation and inhibiting autophagy [26]. On the other hand, autophagy can increase the levels of amino acids after autolysosome-mediated protein degradation. Autophagy effects in immune cells are context-dependent, being associated with both endosomal PRRs activation (TLR7) or degradation (RLRs, NLRP3); enhanced antigen presentation by APCs; memory formation in T cells; T cell stability and survival (including Tregs); impairment of T cell senescence; among others [34, 35]

**Fig. 2:**
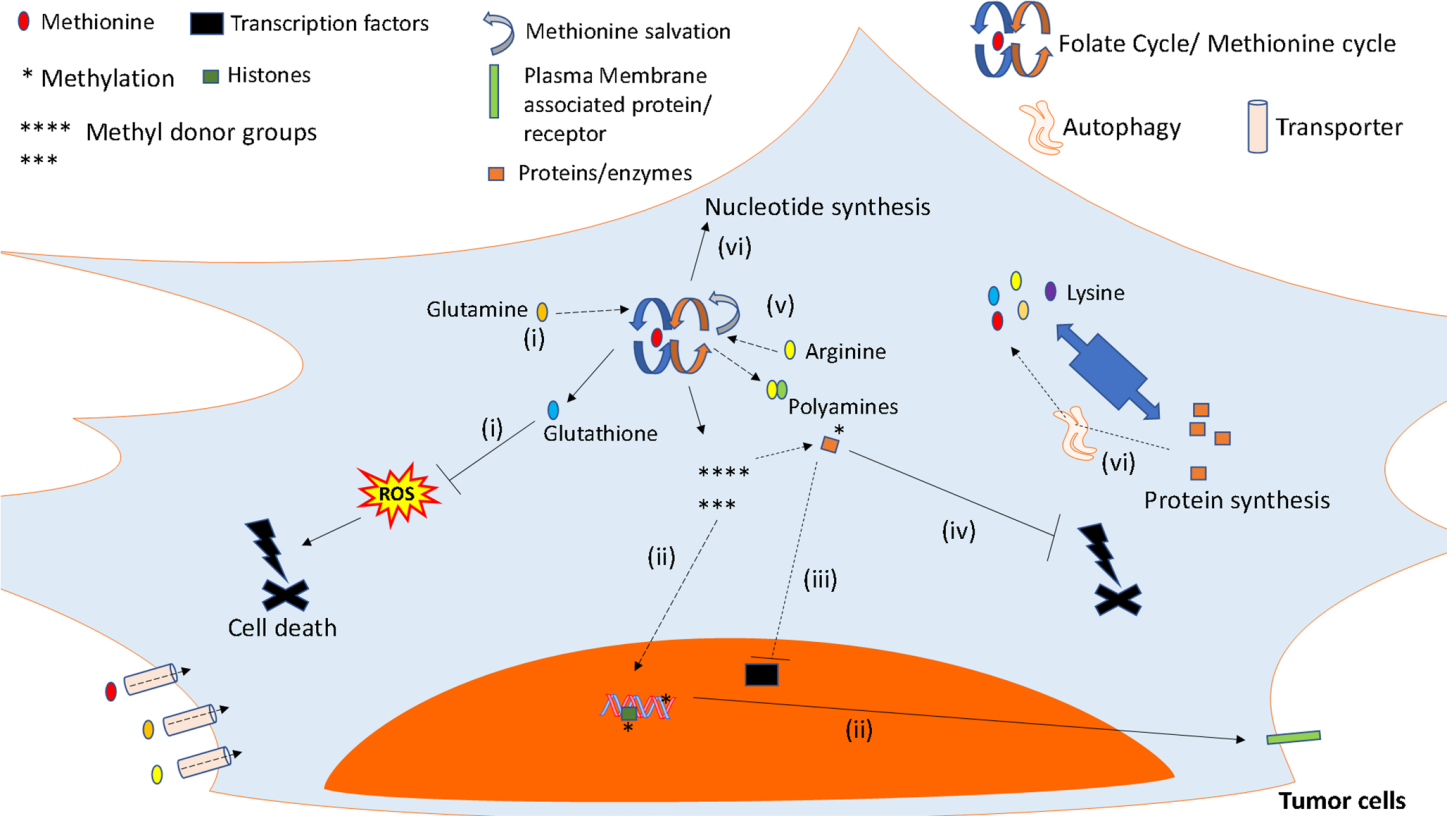
Main mechanisms by which amino acids, focusing on methionine, influence the biology of tumor cells. Amino acids exert a complex role in tumor cells’ biology. (i) Glutamine, cysteine, serine, glycine and methionine are involved in antioxidant responses, promoting the synthesis of glutathione, and being critical for tumor cells survival [117]; (ii) The production of methylation donors by the methionine cycle foster PTMs and epigenetic changes in tumor cells, which can be associated with oxidative phosphorylation and cell survival in the presence of arginine or glutamine [36, 262], and immune escape in the presence of methionine [63]; (iii) methylation also impairs cGAS activation, nuclear translocation of the transcription factors IRF3/7 and NF-kB, subsequent IFN-I release, and expression of MHC-I in tumor cells, leading to antitumor immune responses by CD8 + T cells [55]; (iv) Methylation (associated with methionine cycle) restricts RIPK1 activity, impairing RIPK1-mediated cell death [59]; (v) polyamine synthesis, induced by ranolazine, influences the epigenetic landscape of melanoma cells reducing cellular proliferation, while promoting the expression of PD-L1 [61]; (vi) since tumor cells acquire a highly proliferative state, consumption of amino acids is pivotal for their ability to synthesize macromolecules, especially proteins and nucleic acids. In this context, mTOR activation by amino acids promotes anabolism and restrains catabolic reactions, like autophagy [26]. Thus, arginine-mediated inhibition of autophagy promotes breast tumor cells’ death [93], since, in this context, autophagy is a critical stress-response mechanism. However, some amino acids, like glutamine, can promote autophagy in a mTOR-independent manner, leading to osteosarcoma cells’ survival [190]

Similar to methionine, lysine, another essential amino acid, can be metabolized to promote PTMs associated with epigenetic changes, e.g., crotonylation of histones. Through this, lysine restrains IFN-I signaling and reduces CD8 + T cell activity, also inducing a tolerogenic phenotype in DCs [70] (thereby restricting DCs’ ability to induce Teffs after antigen presentation) [50, 71], probably restricting antitumor immunity. In this context, the deprivation of dietary lysine epigenetically remodels immune cells, effectively inhibiting tumor growth [71]. On the other side, lysine, like all amino acids, supports protein synthesis. In this sense, lysine is critical for T cell-mediated antitumor immunity against hepatocellular carcinoma in a mouse model, maintaining STAT3 levels in T cells [72]. Additionally, lysine deprivation from the TME due to Slc3a2 expression by tumor cells, outcompeting T cells for lysine acquisition, leads to effector T cells (Teffs) inhibition [72]. The role of lysine metabolism in tumor cells is complex. On one side, lysine catabolism promotes acetoacetate generation, which restrains breast tumor cells’ proliferation through the induction of autophagy and senescence [73]. On the other side, lysine protects glioblastoma cells from arginine-derived nitric oxide and lysosomal stress [74]. These results underscore the context-dependent effects of lysine in distinct tumor and immune cells (Table 1; Figs. 1 and 2).

### Threonine

Threonine, like methionine, can be metabolized into SAM and support the folate and methionine cycle, possibly mimicking or complementing at least some of the effects of methionine in tumor biology, discussed above. Furthermore, threonine is a fundamental amino acid for N6-threonylcarbamoyltransferase (YRDC)-enzymatic activity. YRDC mediates the formation of N6-threonylcarbamoyladenosine on tRNAs that decode codons containing adenosine as the first nucleotide. YRDC is critical for the translation machinery of mammalian cells, including immune and tumor cells. In this sense, YRDC-mediated tRNA modifications are involved in tumor growth. Therefore, threonine depletion restrains glioblastoma growth in a mouse model [75]. On the other side, threonine can support SAM generation, inhibiting tumor growth and promoting effector T cells’ functions, through the support of methyl reactions involved in PTMs that affect protein function and the epigenetic landscape/ gene expression of cells [76, 77]. Threonine can also be metabolized into pyruvate and support acetate generation [10], which affects tumor biology in multiple ways, influencing glycolysis, oxidative phosphorylation (Oxphos) and, again (through acetylation), the epigenetic landscape of genes, leading to the expression of ligands of checkpoint receptors (PD-L1) (protumor) or cellular differentiation (antitumor) in different cancers [78, 79]. In summary, the effects of threonine on tumor biology are also context-dependent (Table 2).

**Table 2:** The dual role of threonine in tumor biology (and immune responses)

Amino acid	Protumor effects	Antitumor effects	Therapeutic opportunities
**Threonine**	Supports YRDC-mediated tumor growth in a mouse model of glioblastoma [75]; supports acetate generation and epigenetic changes, leading to immune escape by the expression of PD-L1 in tumor cells [78].	Supports folate cycle and SAM generation, critical for T cells metabolism and function (including antitumor ones) [76, 77].	Specific delivery of threonine to T cells can promote antitumor immunity.

### Arginine

Arginine possesses a dichotomous role in the regulation of immune responses and, consequently, antitumor immunity, also affecting tumor cells biology. Like many other amino acids that serve as carbon and nitrogen sources, arginine is critical for the metabolic fitness and function of immune cells, such as T cells (including memory T cells), macrophages, and NK cells, and tumor cells. Arginine and its byproducts participate directly and indirectly in distinct metabolic pathways, for example, the urea cycle, which can support tumorigenesis [80], and the folate cycle. In macrophages, the arginine role can be determined by the expression of different enzymes in distinct subtypes, i.e., M1 or M2 macrophages, promoting proinflammatory or anti-inflammatory effects through the expression of induced Nitric oxide synthase (iNOS) or Arginases, respectively. Downstream Arginases, glycine amidinotransferase, and guanidinoacetate methyltransferase can generate creatine, which also polarizes macrophages to the M2 phenotype (protumor) [81], but can induce ferroptosis of tumor cells (human fibrosarcoma cells) {antitumor} [82]. This also seems to be true for T cells. Thus, arginine-mediated induction of mTOR seems to restrict Treg cells’ function and promote CD8 + T cells’ antitumor immunity [83]. On the other side, Arginase 2 expression (the mitochondrial isoform) in Tregs promotes mTOR and metabolic fitness of these cells in the skin of mice, enhancing their suppressive function, including in a melanoma model [84]. Once again, the differential metabolism of Teffs (requires mTOR for their activation) and Tregs (mTOR is chronically elevated, being transiently inhibited for their function after Tregs activation) [25] is at the center stage of the antagonistic role of arginine in distinct cells.

Arginase 1 (cytosolic isoform) expression in MDSCs is also important for the suppressive function of these cells, restraining Teffs-mediated antitumor immunity and promoting both Teffs’ cell death and Tregs’ suppressive effects [85, 86]. In a mouse model of breast cancer, arginine depletion in the TME by macrophages, differentiated in the presence of transforming growth factor beta (TGFβ), is associated with the inhibition of CD8 + T cells recruitment, due to the enhanced synthesis of collagen. Arginine consumption also impaired the function of CD8 + T cells due to proline synthesis and ornithine, a non-protein amino acid, secretion. Ornithine (an arginine byproduct) suppressed the metabolism of CD8 + T cells, reducing the intracellular levels of adenosine triphosphate (ATP), and, at the same time, increasing the resistance of tumor cells to CD8 + T cells-mediated cytotoxicity [87]. Also, in another breast cancer mouse model, arginine consumption by tumor-associated macrophages (TAMs) increased the intracellular concentration of the polyamine spermine that promotes epigenetic alterations, i.e., DNA demethylation, in these TAMs. These epigenetic alterations, mediated by p53 signaling and subsequent transcription of PPARγ, led to increased expression of PD-L1 and secretion of IL-10 and TGFβ, which restrained CD8 + T cells activation [88]. Furthermore, arginine depletion impaired T cells proliferation, mediated by GCN2 [19], and signaling downstream TCR due to downregulation of CD3ζ [89, 90]. NK cells’ antitumor activity also seems to depend on arginine levels, at least in an animal model based on human gastric cancer cells [91].

In a mouse model of colorectal cancer, increased arginine levels in the TME promotes tumor development through (i) enhanced angiogenesis due to arginine metabolism and synthesis of the proangiogenic gas nitric oxide (NO) by cells expressing endothelial nitric oxide synthase (eNOS); (ii) a rise in the levels of putrescine, a derivative metabolite from arginine (urea cycle), and the chemokine CCL2 leading to M2 macrophages differentiation and the impairment of antitumor immunity; and (iii) eNOS-mediated arginine catabolism leading to upregulation of wingless/integrated (Wnt) pathway and accelerated progression of colorectal cancer [92]. Differently, in a mouse model of myeloma, arginine depletion by arginase-expressing M2 macrophages is associated with tumor cells starvation and death [18]. Furthermore, the depletion of arginine restrains Oxphos in the mitochondria, after epigenetic changes (i.e., reduced histone acetylation due to a restricted pool of acetyl-CoA) in tumor cells (breast cancer), impairing tumor development. This antitumor effect could be reversed by aspartate supplementation [26], reinforcing that the presence of other metabolites (in this case, another amino acid) in the TME can influence the effects of a single one in tumor biology. Intriguingly, arginine-mediated inhibition of autophagy promotes breast tumor cells death in the presence of inhibitors of heat shock protein 70 (a stress-response protein involved in the adaptation to proteostasis) [93]. Furthermore, IFNγ-induced arginine depletion, subsequent GCN2 expression, and autophagy contribute to the transformation of bovine epithelial mammary cells and tumorigenesis [94]. In addition, sustained arginine deprivation in Argininosuccinate Synthase 1 (ASS1)-deficient melanoma and breast tumor cells promotes autophagy, but, in this scenario, autophagy is associated with tumor cells death [95, 96]. ASS1 is a critical enzyme for the urea cycle and biosynthesis of arginine. These context-dependent effects of cellular processes, transcription factors, and metabolites are present in many aspects of biology, influenced by the concomitant expression of receptors, metabolic enzymes/pathways of each cell, molecular partners, and convergent or divergent signaling/ transcription factors activation.

These studies underscore the paradoxical role of arginine metabolism and availability depending on the circumstances (e.g., immune cells infiltration and the type of arginine metabolizing enzymes expressed in these cells in the TME) and type of tumor, implying that arginine supplementation might be interesting for some, but not other tumors (Table 3). An interesting therapeutic strategy is to induce arginine transporters (e.g., Slc7a2) in antitumor T cells, before their adoptive transfer to patients, enhancing their ability to compete for arginine in the TME, without the deleterious effects of arginine depletion for antitumor immunity [8]. Arginase and NO modulators are also compelling pharmaceuticals to be explored against tumors. The enzymes involved in the metabolism of arginine, mainly arginase 1 and 2 or NOS, affect antitumor immune responses, as previously discussed, but also tumor and stromal cells metabolism. In this context, arginase 1 expression is important for polyamine and proline synthesis by tumor cells and cancer-associated fibroblasts (CAFs), providing them (CAFs) with the carbon backbone for collagen and ECM synthesis that fosters ovarian tumor growth and metastasis in a mouse model [97]. Thus, arginase 1 inhibitors can exert a double-strike, promoting antitumor immunity and dysregulating important metabolic pathways for tumor and CAFs cells biology/function.

**Table 3:** The dual role of arginine in tumor biology (and immune responses)

Amino acid	Protumor effects	Antitumor effects	Therapeutic opportunities
**Arginine**	Increases tumor resistance to cytotoxicity (in a murine model of breast cancer) [87]; promotes DNA demethylation, repressing CD8 + T cell activation (in a mouse model of breast cancer) [89]; promotes tumor development, inducing Wnt pathway, and angiogenesis (in a mouse model of colorectal cancer) [94]; restrains antitumor immunity (in a mouse model of melanoma) [84]; supports tumor cells (myeloma and breast cancer) metabolism [18, 26]; its degradation by Arginase1 restrains antitumor immunity through different mechanisms, including the synthesis of polyamines, inhibition of T cells fitness (restrains aerobic glycolysis) and MDSCs suppressive functions [85–87, 89].	Restricts the suppressive functions of Tregs, and stimulates antitumor immunity of CD8 + T cells, after the induction of mTORC1 [83]; it is critical for the metabolic fitness and function of immune cells, such as T cells (including memory T cells) [19, 89, 90]; promotes nitric oxide production by macrophages, which can promote tumor cells death.	Specific delivery of arginine to Tregs and CD8 + T cells can promote antitumor immunity, especially if combined with inhibitors of DNA methyltransferases. In the TME, arginine depletion can impair the resistance of tumor cells to cytotoxicity. Inhibition of the pathways involved in polyamine synthesis can enhance antitumor immunity in some situations. Arginase 1 can be targeted to improve antitumor immunity and impair tumor support by the ECM.

### Proline

Proline, a nonessential amino acid with a distinctive secondary amine group, is an important component of extracellular matrix (ECM) proteins. In this sense, proline metabolism in cancer-associated fibroblasts (CAFs) is critical for the synthesis of ECM that supports tumor growth and metastasis [98]. For several tumor cells, proline also seems to be important for their metabolism, and its synthesis is associated with tumor progression [99, 100]. In this sense, since arginine can promote proline synthesis, low arginine levels might be associated with resistance to tumor development. Interestingly, the transcription factor c-Myc (involved in tumorigenesis in several cancers) can both promote proline (and also serine [101]) synthesis and inhibit proline breakdown [102], contributing to the protumor effects of proline. In tumor cells, proline also supports antioxidant responses, contributing to cell survival, and ROS-dependent NF-kB expression and secretion of IL-4, IL-6, and IL-13, which promote the immune suppressive M2 phenotype in macrophages [102, 103]. On the other hand, proline metabolism supports chimeric antigen receptor (CAR) T cells’ antitumor activity against breast cancer cells [104], and also the proinflammatory effects of macrophages, supporting trained immunity [105] (Table 4).

**Table 4:** The dual role of proline in tumor biology (and immune responses)

Amino acid	Protumor effects	Antitumor effects	Therapeutic opportunities
**Proline**	Supports the metabolism of tumor cells [99, 100] and the secretion of cytokines involved in M2 (protumor) macrophages polarization [101].	Promotes the antitumor activity of CAR T cells [104]; supports trained immunity in macrophages [105], which can contribute to antitumor immunity [261].	Specific delivery of proline to TAMs and T cells can promote antitumor immunity.

### Serine and Alanine

Serine is a non-essential glucogenic amino acid. Serine can be converted to glycine by serine hydroxymethyltransferase [106], being important for different metabolic pathways, such as sphingolipids, nucleotide, and glutathione (antioxidant intracellular responses) biosynthesis and folate cycle (one-carbon metabolism) [106–108]. Not surprisingly, serine metabolism differently affects immune responses and tumor biology. Serine is crucial for Teffs’ metabolism, and its deprivation impairs their functions, since they rely on exogenous serine [109]. Interestingly, serine is also critical for the metabolism of Tregs, and a serine-free diet can lead to reduced levels of Tregs in the TME, restraining melanoma growth in a mouse model [110]. In contrast, serine-induced mTOR inhibits Tregs’ suppressive activity (impeding Foxp3 expression), but this can be reversed by serine degradation to glutathione [110], reinforcing that the manner by which cells metabolize some amino acids can influence their function, being a target for therapies. Serine also restricts aberrant cytokine secretion by macrophages [111], promoting mitochondrial homeostasis, controlling oxidative stress and inflammation in aged mice [112], possibly suppressing tumorigenesis during senescence. D-serine, an isomer of serine (L-serine), can restrict CD8 + T cells responses through the inhibition of the transcription factor T-box expressed in T cells (T-bet), restraining IFNy production. Reduced levels of IFNγ are associated with an inefficient antimycobacterial and, possibly, antitumor immunity [113]. This was mediated by WD repeat-containing protein 24 (WDR24) expression in T cells, and as D-serine can also be present in the TME, it is also possibly involved in the silencing of T cells’ antitumor immune responses.

These studies underscore that the effects of serine might depend on the type of infiltrating immune cells (Tregs or Teffs) and their specific isomer, being an interesting target for supplementation in tumors with a high ratio of infiltrated Teffs/Tregs. In tumor cells, serine is critical to maintain proliferation, contributing, for example, to the synthesis of nucleotides [114]. Therefore, impeding serine biosynthesis or acquisition, or both, is an interesting molecular target against specific tumors [114]. Accordingly, inhibitors of enzymes involved in serine biosynthesis have been tested against tumors, both as single or adjuvant therapies. In this sense, Ma et al. [115] described that phosphoglycerate dehydrogenase (PHGDH), involved in the synthesis of serine and glycine from glucose, is critical for pancreatic tumor cells proliferation and tumorigenesis, mediating cell-tight junctions formation, mTOR activation (through Akt), and protein expression and translation, after interaction with the translation initiation factors eIF4A1 and eIF4E (which seems to be independent of its enzymatic activity). PHGDH-mediated biosynthesis of serine is also essential for M2 macrophages’ metabolism, promoting α-ketoglutarate synthesis and mTOR activity. Genetic ablation of PHGDH led to a phenotypic shift of M2 macrophages to M1, and antitumor T-cell mediated immunity against mesothelioma cells in a mouse model [116]. Interestingly, PHGDH also affects tumor biology independently of its effects in serine metabolism, for example, directly interacting with the RNA-binding protein Poly(rC)-binding protein 2 (PCBP2), and inhibiting its proteasomal degradation. Then, PCBP2 stabilizes Slc7a11, involved in the import of cysteine that is crucial for the synthesis of glutathione and antioxidant responses, preventing bladder cancer cells from ferroptosis [117]. Another important enzyme for serine/glycine metabolism is Serine Hydroxymethyltransferase (SHMT), which mediates the reversible conversion of serine to glycine and one-carbon metabolism. The two isoforms of SHMT (SHMT1 and SHMT2) affect tumor biology. SHMT1, but not SHMT2, is critical for serine and glycine metabolism, supporting ATP generation in human lung cancer cells. In this context, SHMT1 knockdown leads to impaired migratory ability, due to increased ROS levels, reduced ATP generation, and AMPK activation, affecting cytoskeleton remodeling [118]. SHMT1-mediated metabolism also influences the ability of tumor cells to synthesize crucial lipids, like sialic acid N-acetylneuraminic acid, which supports the release of cytokines (IL-6 and IL-8) involved in ovarian cancer cells’ proliferation and migration in mouse models [119]. SHMTs are also involved in antioxidant responses, through one-carbon metabolism, of human lung cancer cells, being critical for their survival [120]. SHMT2 also exerts influence on tumor cells’ ability to migrate and proliferate, supporting metabolic adaptations through Akt2 and n-Myc activation in human neuroblastoma cells [121], or hypoxia inducible factor 1 (HIF-1) activation in human gastric cancer cells [122]. In conjunction, these studies underscore the important effects of enzymes involved in serine metabolism (also reviewed very competently by [27]) in tumor cells biology, being targets for inhibition. However, PHGDH and SHMTs are also important for immune cells metabolism and function, at least against fungi and viral infections [123, 124], respectively, and the specific delivery of inhibitors to tumor cells might be the best therapeutic alternative in this case, not affecting immune cells-mediated antitumor immunity. It is also important to bear in mind that liver SHMT2 mainly mediates the consumption of glycine (which can be converted to serine), regulating (reducing) its levels in circulation, which can also be targeted to improve antitumor therapies associated with glycine/serine [125] (Table 5).

**Table 5:** The dual role of serine and alanine in tumor biology (and immune responses)

Amino acids	Protumor effects	Antitumor effects	Therapeutic opportunities
**Serine**	Supports Tregs and tumor cells’ metabolism [110, 116].	Supports Teffs’ metabolism and function [111]; induces mTOR activation, impairing Tregs’ function, depending on the expression of the distinct enzymes that degrade serine [110].	Specific delivery of serine to T cells can promote antitumor immunity. Specific inhibition of enzymes involved in serine-mediated epigenetic changes can impair tumor cells proliferation.
**Alanine**	Promotes tumor cell survival under stress [10]; supports citric acid cycle, amino acids and lipid biosynthesis in tumor cells, contributing to their proliferation [116].	Supports Teffs’ activation and macrophage-mediated phagocytosis [119, 120].	Specific delivery of serine to tumor cells can promote antitumor immunity.

The de novo biosynthesis of sphingolipids, catalyzed by serine palmitoyltransferase, connects serine and mitochondrial alanine metabolism to membrane lipid diversity, a mechanism that could be exploited to induce tumor cell death under metabolic stress, such as in an amino acid-deprived microenvironment [126]. Moreover, other studies also demonstrate the role of alanine in the TME. In pancreatic ductal adenocarcinoma, alanine is utilized by stroma-associated pancreatic stellate cells to fuel the citric acid cycle, as well as non-essential amino acids and lipid biosynthesis in tumor cells, being protumor in this context [87]. In this microenvironment, specific transporters, such as Slc1a4 and Slc38a2, play an essential role in alanine exchange, being targets for antagonistic therapies [127]. In contrast, the crucial role of alanine in T cells activation and macrophage-mediated phagocytosis can possibly support the function of Teffs cells against distinct tumors and the antitumor role of macrophage-mediated phagocytosis against tumor cells that detached from the tumor mass [128, 129].

### Tryptophan

The role of the amino acid tryptophan in tumor biology is complex. It is critical for T cell metabolism and function [130], and can be degraded into different molecules, such as indole-propionic acid (IPA), indole-3-acetic acid (IAA), and trans-3-indoleacrylic acid by microorganisms, or kynurenine by our own cells (expressing the enzymes indoleamine dioxygenase 1-IDO1-, IDO2, and Tryptophan 2,3-dioxygenase- TDO2). IPA can optimize immunotherapies based on antagonistic anti-Programmed Cell Death Protein 1 (PD-1), enhancing chromatin accessibility and CD8 + T cells stemness [131]. Differently, trans-3-indoleacrylic acid is involved in colorectal carcinogenesis, inhibiting ferroptosis of tumor cells in an aryl hydrocarbon receptor (Ahr)-dependent mechanism [132]. IAA can also promote tumor growth in some cases (e.g., in renal and esophageal cancer cells), for example, by inducing Ahr in macrophages and Teffs, suppressing antitumor immunity [133]. In contrast, depending on its concentration, IAA inhibits neuroblastoma cells, gastric cancer cells, lung cancer cells and ovarian cancer cells proliferation, through different mechanisms, including activation of Toll-like receptor 4 and MAPKs signaling [133, 134]. Kynurenine can inhibit Teffs’ function through distinct mechanisms as well, including the activation of Ahr after being transported by Slc7a11 [130], which is also involved in the acquisition of cystine that leads to antioxidant responses and invasiveness of melanoma cells in a mouse model [135]. Interestingly, kynurenine can both inhibit or promote tumor cells proliferation depending on the tumor cell type. For example, kynurenine restrains melanoma and endothelioma cellular proliferation [136, 137], impairing DNA synthesis and inducing necrosis in melanoma cells, while promoting the β-catenin pathway and colon cancer cells multiplication in a dose-dependent manner [138]. In addition, kynurenine, as an activator of Ahr, is involved in clonogenic survival and motility of glioma cells, probably contributing to metastasis [139].

Kynurenine can be further degraded into different metabolites, like quinolinic acid (degraded to nicotinamide adenine dinucleotide- NAD + ) and picolinic acid, which have distinct effects. For instance, NAD+ possesses anti-inflammatory functions (inhibiting NLRP3 [140]), while picolinic acid activates the proinflammatory effects of macrophages [141]. Thus, the outcome associated with tryptophan degradation might also depend on the enzymes involved in its breakdown and the breakdown of its derivatives, like kynurenine.

Ahr effects, induced after kynurenine import, in tumor biology can also be dichotomous, depending on the tumor type and the infiltrated immune cells, increasing the complexity of the discussion about the tryptophan metabolism in tumor biology. For example, though Ahr can inhibit CD4 + T cells’ function and support Tregs differentiation [142], it promotes the antimicrobial activity of macrophages and both CD8 + T cells and NK cells-mediated cytotoxicity [132, 135, 143–145]. In tumor cells, Ahr can inhibit both lung tumor cells’ invasion and glioblastoma cells’ growth [146, 147] but promote ovarian cancer cells’ proliferation and metastasis [148].

**Fig. 3:**
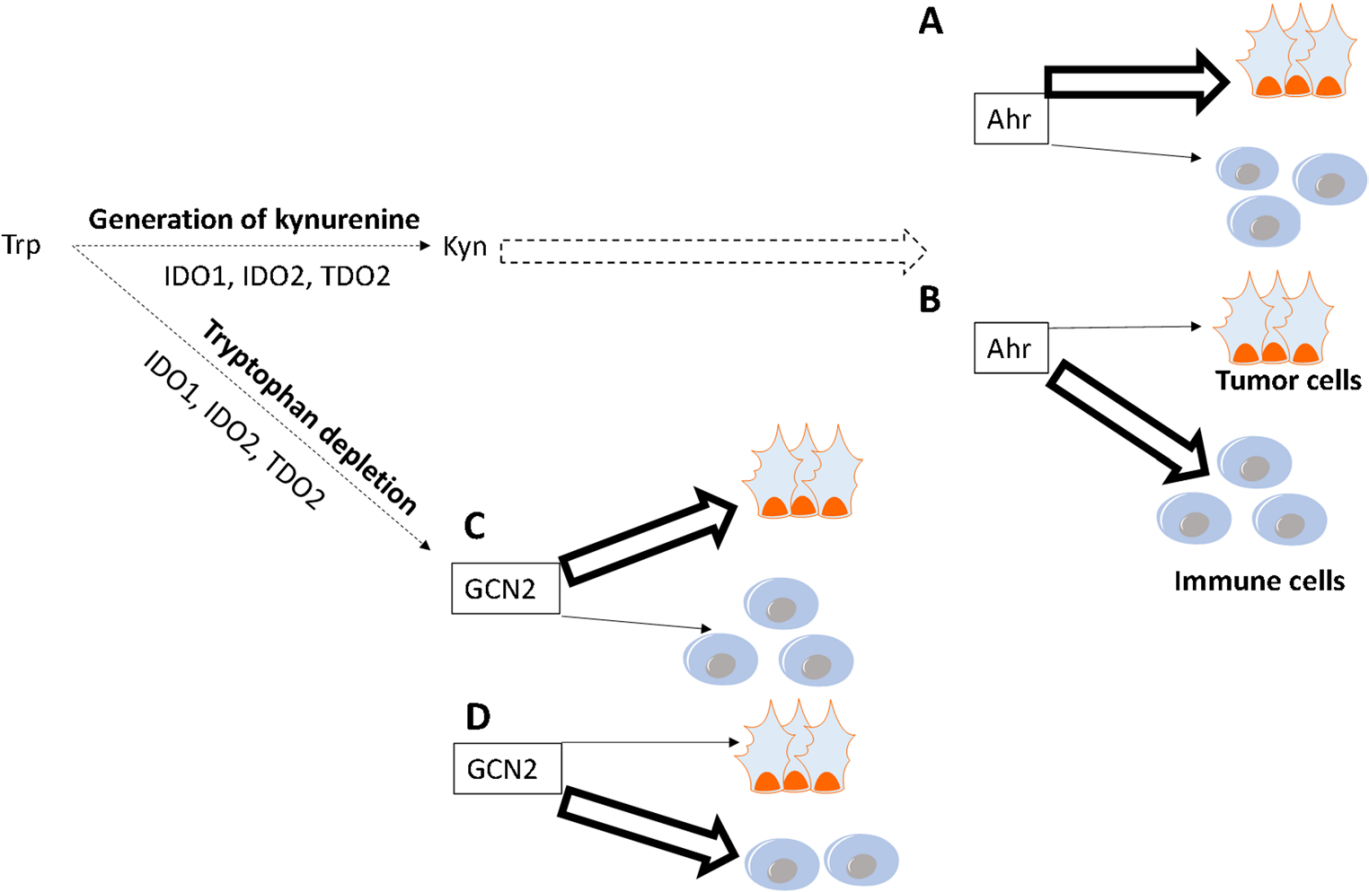
The complex role of tryptophan metabolism in tumor biology: Tryptophan is a fundamental amino acid for both tumor and immune cells. Tryptophan can be degraded by distinct enzymes, i.e., IDO1, IDO2, and TDO2, expressed by both immune and tumor cells. These enzymes lead to the generation of kynurenine, which can be further degraded into other metabolites. Kynurenine can be transported to the intracellular compartment of immune cells or tumor cells, by Slc7a11, for example, depending on which cells express higher levels of the kynurenine transporters. A) In the cytoplasm, kynurenine promotes Ahr activation. Like many other transcription factors, the effects of Ahr are complex, depending on the presence of other molecular partners and the epigenetic landscape of each specific cell. Therefore, Ahr can lead to the inhibition or enhancement of tumor cells proliferation [146–148]. B) The acquisition of kynurenine by immune cells and subsequent activation of Ahr can also lead to distinct outcomes, depending on the type of immune cells. Thus, kynurenine can promote the effector functions of CD8 + T cells, macrophages, and NK cells, but inhibits the effector functions of CD4 + T cells, also leading to the differentiation of CD4 + T cells into a suppressive phenotype [142–145]. C) Concomitantly, tryptophan depletion exerts a nutritional stress in the cells from the TME, leading to the expression of GCN2, a signaling kinase that coordinates stress responses. Similar to Ahr, GCN2 also affects, in a complex way, both tumor and immune cells. In tumor cells, GCN2 expression can promote cell proliferation and survival, but also apoptosis, depending on the context [268]. D) In immune cells, GCN2 promotes the effector functions of CD8 + T cells, but is associated with CD4 + T cells’ anergy and the suppressive function of myeloid cells [151–154]. The ability of specific immune cells or tumor cells to adapt to the TME (for example, by acquiring other amino acids or nutrients) in the absence of tryptophan will determine the levels of expression of GCN2. The role of GCN2 will depend on the expression of its molecular partners and the epigenetic landscape of each cell. In addition, the type of infiltrated immune cells also influences how antitumor immunity will be affected by both Ahr and/or GCN2 activation

Because tryptophan is an important carbon source, its extracellular degradation/ consumption imposes a metabolic challenge to both immune and tumor cells from the TME. Therefore, these cells must express GCN2 (general control nonderepressible 2) to adapt to tryptophan and other amino acids, like arginine, deprivation [149]. GCN2 promotes stress-response mechanisms, like autophagy, amino acid uptake, and the secretion of vascular endothelial growth factor (VEGF) with subsequent induction of angiogenesis for the acquisition of nutrients [150]. Compellingly, GCN2 expression in CD4 + T cells is associated with anergy and tolerance to tissue (tumor) damage [151, 152], probably being an interesting molecular target for antagonism, improving antitumor immunity while also restraining tumor cells survival, at least for some tumors. Additionally, GCN2 expression in macrophages and MDSCs promotes their suppressive function, restraining antitumor immunity in mouse models of colon cancer and melanoma [153]. On the other hand, GCN2 can be crucial for CD8 + T cells’ antitumor immunity against gliomas [154], probably through metabolic adaptations and resistance to cell death. Therefore, GCN2 expression in tumor cells, CD4 + T cells, and myeloid cells is associated with a protumor effect, promoting tumor cells’ survival and restricting antitumor immunity. Contrarily, GCN2 expression on CD8 + T cells promotes inflammatory responses and antitumor immunity, meaning that the presence of specific tumor-infiltrated immune cells might be critical for the outcomes related to GCN2 expression. Furthermore, in the absence of tryptophan, neoantigens generated by the substitution of this amino acid for phenylalanine in proteins can be targeted by T cell-mediated immune responses [155]. Thus, in these two contexts above, tryptophan depletion might lead to tumor regression. Another confounding feature, especially in transcriptomic analysis of tumors, is that IDO1 expression is increased in M1 macrophages [156], probably as a means to fine-tune the tissue-destructive effect of these cells. In this context, IDO1 expression might be associated with antitumor immune responses, as a marker of M1 macrophages, but not related to the enzymatic and anti-inflammatory activity of IDO1. In conclusion, tryptophan metabolism can be a compelling target for antitumor therapies, depending on both the tumor and immune cells from the TME (Table 6; Fig. 3).

**Table 6:** The dual role of tryptophan in tumor biology (and immune responses)

Amino acid	Protumor effects	Antitumor effects	Therapeutic opportunities
**Tryptophan**	Trans-3-indoleacrylic acid (a tryptophan byproduct) promotes colon cancer cells proliferation [132]; Kynurenine (a tryptophan byproduct) contributes to clonogenic survival and motility of glioma cells [139], and proliferation of colon cancer cells [138]; promotes ovarian cancer cells proliferation and metastasis [148]; its depletion promotes inflammatory responses and antitumor immunity in GCN2-expressing CD8 + T cells and macrophages [154, 155]	It is critical for T cells metabolism and function [130]; kynurenine restrains cellular proliferation in melanoma and endothelioma [136, 137]; Tryptophan depletion promotes angiogenesis in tumor cells expressing GCN2 [150]; GCN2-expressing CD4 + T cells (after tryptophan depletion) are associated with anergy and (tumor) tissue damage tolerance [151, 152].	IDO1 can be induced for tryptophan depletion in the TME and a better control of tumor growth due to GCN2-expressing CD8 + T cells-mediated antitumor immunity, or kynurenine-mediated Ahr expression and inhibition of tumor cells proliferation (glioblastoma and lung tumor cells). On the other hand, IDO1 can be inhibited to impair kynurenine-mediated silencing of immune cells and tumor cells proliferation (ovarian cancer cells). Combining tryptophan depletion and GCN2 inhibition can impair tumor cells’ adaptation to nutritional stress, leading to cell death, but it might also restrain antitumor immunity mediated by CD8 + T cells.

### Branched-Chain Amino Acids

The role of branched-chain amino acids (BCAAs), which include leucine (ketogenic), isoleucine, and valine (glucogenic ones), in tumor biology and immune responses is being described in different studies (Table 7). BCAAs can promote cellular anabolism, fueling the citric acid cycle and promoting the biosynthesis of proteins and lipids, through mTOR activation, which correlates to tumor aggressiveness in a mouse model of hepatocellular carcinoma [157]. They can also be associated with proliferation and tumorigenesis in a mouse model of colorectal cancer through sonic hedgehog signaling [158]. Furthermore, valine can inhibit genomic instability through its interaction with histone deacetylase 6 (HDAC6) in the cytoplasm. In the absence of valine, HDAC6 accumulates in the nucleus and promotes Tet2 (ten-eleven 2) deacetylation, subsequent DNA demethylation, and genomic instability in cancer cells [159]. In this context, valine restriction can accentuate the antitumor activity of Poly ADP ribose polymerase (PARP) inhibitors, since PARP mediates DNA repair and tumor cells survival [159]. Additionally, lowered BCAAs availability and/or inhibition of valine degradation in prostate cancer cells diminished lipid metabolism. Consequently, there was a selective reduction in malignant prostate cell proliferation, a decrease in intracellular succinate levels, and compromised cellular respiration [160]. In contrast, BCAAs exert an antitumor effect in mouse models of breast tumors [161], probably by promoting cellular oxidative stress after autophagy, which is involved in pancreatic tumor cells’ survival [162]. BCAAs also enhance the antitumor (mouse model of lung cancer) activity of CD8 + T cells, through the optimization of glucose metabolism and uptake by these cells, supporting their release of IFNγ, tumor necrosis factor (TNF), and granzyme B [163]. Leucine, a pure ketogenic amino acid (along with lysine) can also regulate immune cells’ function, influencing antitumor immunity in a dichotomous manner. On one side, leucine promotes T cells and B cells’ responses [164, 165], which are critical for antitumor immunity. On the other side, leucine and glutamine transport by Slc7a8 induces CD47 expression by tumor cells and immune escape in a mouse model of osteosarcoma [166, 167]. Moreover, leucine supplementation in a murine model of pancreatic cancer is related to cancer growth [168], being especially important for melanoma metabolism as well [14]. Leucine promotes mTOR activation, being an inhibitor of autophagy. As discussed by others, the role of autophagy in both tumor and immune cells can be paradoxical [34, 169], which adds another layer of complexity for the role of leucine in tumor biology.

**Table 7:** The dual role of BCAAs in tumor biology (and immune responses)

Amino acids	Protumor effects	Antitumor effects	Therapeutic opportunities
**BCAAs**	Promote cellular proliferation and tumorigenesis in a mouse model of colorectal cancer [158]; contribute to tumor aggressiveness in hepatocellular carcinoma [157]; valine promotes cell survival, inhibiting genomic instability [159]; promote lipid metabolism in prostate cancer cells; Leucine uptake induces CD47 expression by osteosarcoma cells and immune escape by binding to the inhibitory receptor SIRPα in myeloid cells [166, 167].	Promote cellular oxidative stress after autophagy in pancreatic tumor cells [162]; enhance the antitumor activity of CD8 + T cells [163]; leucine improves B cells’ secretion of IgG [164].	Specific delivery of leucine to CD8 + T cells can improve antitumor immunity. Combining ROS-inducing agents with BCAAs can enhance tumor cells death.

### Glutamine and Glutamate

Glutamine is an important glucogenic amino acid that can fuel the citric acid cycle for ATP synthesis. The role of glutamine in tumor development and biology is highly influenced by the tumor type, its metabolic requirements, and immune responses, depending on the infiltrated immune cells. Glutamine is imported into cells by distinct carriers from the families of Slc1, Slc6, Slc7, and Slc38 transporters, serving as an essential carbon and nitrogen source for many of these cells, including tumor cells (e.g., pancreatic ductal adenocarcinoma [170]), breast cancer cells [171], and immune cells. Through this simple assumption, glutamine plays a dichotomous role in antitumor immunity, being also involved in many aspects of tumor and immune cell biology, which underscores its complexity in tumor biology, and the uniqueness of certain molecular mechanisms associated with each tumor. For example, glutamine uptake is correlated to CD47 expression and consequent inhibition of macrophage-mediated phagocytosis of tumor cells. This is mediated by the interaction of CD47 with the inhibitory macrophage receptor, SIRPα (Signal regulatory protein alpha) [166]. Glutamine uptake by Slc7a8 (also known as L-type amino acid transporter 2- LAT2) promotes glycolysis, after mTOR activation, and chemoresistance of pancreatic tumor cells in a mouse model [172]. In addition, enhanced glucose and glutamine metabolism support the growth of breast tumor cells, mediated by IL-4 receptor α [171]; and glutamine consumption promotes endometrial tumor cell death, inhibiting autophagy (a stress response mechanism in this context), after estrogen incubation and c-myc activation [173].

c-Myc, already cited above, regulates glutamine metabolism in human lymphoma cells and prostate cancer cells by increasing the expression of mitochondrial glutaminase. This enzyme converts glutamine into glutamate, which is then used for both ATP production through the citric acid cycle or glutathione synthesis, ultimately supporting cell proliferation and maintaining ROS homeostasis [174]. Glutamine metabolism can also promote PTMs in proteins, including glutamylation, O-GlcNAcylation (glucosamine linked to serine or threonine residues in proteins) and N-linked glycosylation (sugar residues attached to asparagine residues in specific proteins). Abnormal patterns of N-glycosylation and O-GlcNAcylation are commonly found in tumor cells and can also affect the functions of immune cells [175]. Glutamylation of viral proteins (human immunodeficiency virus- HIV) can restrain the activation of STING and subsequent IFN-I production by infected/immune cells [176]. As discussed elsewhere, the role of IFN-I in tumor biology and immune responses is, per se, dichotomous, promoting cytotoxicity of NK cells and CD8 + T cells in some circumstances but also the suppressive function of Tregs in others [177–179].

Glutamine promotes tumor development in many distinct manners, such as (i) supporting epigenetic modifications in cellular FLICE-like inhibitory protein (cFLIP) and resistance of pancreatic tumor cells to apoptosis [93]; (ii) glutamine-derived aspartate supports eukaryotic translation initiation factor 5 A (EIF5a) hypusination and HIF1α translation, promoting metabolic changes, i.e., enhanced glycolysis, in tumor-associated macrophages, which is correlated to advanced tumors [180]; (iii) glutamine promotes MDSCs recruitment and suppressive function, while inhibition of glutamine metabolism induced proinflammatory myeloid cells with antitumor activity in a mouse model of breast tumor [181]; accordingly, glutamine synthetase (GS), which promotes glutamine synthesis from glutamate, restrains both M1 macrophage differentiation and antitumor immunity, and its inhibition promotes implanted lung tumors elimination in mice [50, 182]; (iv) glutamine polarizes macrophages to M2 phenotype [183] and, as a precursor of glutamate, can also restrain macrophages function, after glutamate-induced N-methyl-D-aspartate receptor (NMDAR) channel activation, leading to tumor development in mouse models of fibrosarcoma and hepatocellular carcinoma [184]. In addition, tumor cells-derived glutamate promotes aspartate synthesis by cancer-associated fibroblasts, which fuels tumor cells’ proliferation, supporting the biosynthesis of nucleotides [185]. On the other hand, glutamate can promote tumor cells death (apoptosis), which is counter-regulated by autophagy [186]; (v) glutamine also promotes atypical memory B cells generation in tertiary lymphoid organs associated with tumors [172]. These atypical memory B cells possess immune regulatory functions, for example, expressing PD-L1, and IL-10, and produce low-affinity antibodies [187]; (vi) glutamine can promote the synthesis of GABA that stimulates the β-catenin pathway, through its receptor, in tumor cells, fostering cellular proliferation (of tumor cells) and impairing CD8 + T cells antitumor activity [188]; (vii) glutamine consumption by clear cell renal cell carcinoma in vitro leads to IL-23 secretion by macrophages and Tregs recruitment, possibly restraining antitumor immunity in humans too [189]; (viii) glutamine-derived ammonia promotes osteosarcoma cell survival through the induction of mTOR-independent autophagy [190].

On the other hand, glutamine metabolism can be associated with tumor elimination. Glutamine is critical for (i) NK cells [191] and Teffs responses, especially Th17 cells and CD8 + T cells, while its absence is associated with Treg cells development in vitro, under Th1-inducing conditions [192–194]; (ii) glutamine promotes the production of lactate and antitumor (mouse melanoma model) immune responses by macrophages [195]; (iii) glutamine starvation leads to granulocyte colony stimulating factor (G-CSF) and granulocyte macrophage colony stimulating factor (GM-CSF) expression and subsequent MDSCs development [196], implying that glutamine supplementation might enhance antitumor immunity after the impairment of MDSCs development; (iv) glutamine, as a source of glutathione, promotes resistance against immune suppression mediated by MDSCs-derived methylglyoxal in melanoma mouse models [197]. Methylglyoxal is a reactive dicarbonyl molecule that can be generated from metabolites originating in glycolysis (dihydroacetone phosphate), lipolysis (acetol), and amino acid degradation (aminoacetone). Apart from its immune suppressive effects in CD8 + T cells [197], methylglyoxal also promotes TAMs polarization and breast, colon, and liver tumor cells growth [198]. On the other side, methylglyoxal-induced p53 might restrain tumorigenesis, at least in human endothelial cells [199]. In summary, glutamine supplementation might be a compelling strategy against some tumors with infiltrated CD8 + T cells, but not others in which it is involved directly or indirectly in tumorigenesis. Furthermore, targeting what impairs glutamine to promote its anti-tumor effects can ameliorate therapies, for example, enhancing the expression of glutamine transporters in CD8 + T cells to improve glutamine consumption by these cells in comparison to tumor cells, fostering antitumor immunity.

Targeting the enzymes involved in glutamine biosynthesis/degradation can also be an interesting therapeutic strategy against distinct tumors. In this context, glutaminase 1 (GLS1), expressed in the mitochondria of several malignant tumors (ovarian cancer, breast tumor cells, colorectal cancer), promotes glutamine degradation to support cellular proliferation [200–202]. Therefore, GLS1 inhibitors (especially CB-839) have been described to restrain tumor cells’ proliferation, and also, due to a higher availability of interstitial glutamine, support the antitumor effects of CD8 + T cells, increasing their infiltration and reducing Tregs [203, 204]. Interestingly, the other mitochondrial enzyme (GLS2) involved in glutamine degradation impairs tumorigenesis, being frequently downregulated in tumors. There are several differences in their expression and function. GLS1 is more ubiquitously expressed, while GLS2 is expressed mainly in the brain, liver, and pancreas. Unlike GLS1, GLS2 is not inhibited by CB-839 or glutamate (its immediate byproduct), and can directly bind and inhibit Rac1 (Ras-related C3 botulinum toxin substrate 1), suppressing murine and human hepatocellular carcinoma cells migration and metastasis in a mouse model [205]. Furthermore, GLS2, but not GLS1, is a target of p53, a tumor suppressor protein. Thus, GLS2 can promote glutathione synthesis, improving antioxidant responses [206], being targeted for activation against tumors that thrive on ROS-mediated DNA damage and chromatin instability. Indeed, it has been described that GLS2-knockout mice develop B-cell lymphomas and hepatocellular carcinomas [207]. GLS2 (but not GLS1) can suppress tumorigenesis by increasing lipid ROS production after the facilitated conversion of glutamate to α-ketoglutarate, leading to ferroptosis [207]. The enzyme involved in glutamine synthesis, GS, is also involved in tumor biology. As expected, due to the mainly protumor role of glutamine, several tumor cells rely on GS to maintain their metabolic requirements and proliferative ability. Therefore, different studies demonstrate that GS inhibition/downregulation can restrain tumor growth and resistance to radiotherapy and chemotherapy, suppressing glutamine-dependent nucleotide synthesis [5, 208–212]. Regarding the role of GS in immune cells, as discussed above, it can be dichotomous, since glutamine can promote or hamper, depending on the cells and context, antitumor immunity [166, 172, 182, 192–194] (Table 8).

**Table 8:** The dual role of glutamine in tumor biology (and immune responses)

Amino acid	Protumor effects	Antitumor effects	Therapeutic opportunities
**Glutamine**	Supports pancreatic and breast cancer cells growth [170, 171]; its uptake promotes CD47 expression in macrophages (restraining their activation for antitumor immunity), and resistance to chemotherapy by pancreatic tumor cells [166, 172]; enhances the metabolism and proliferation of breast tumor cells [171]; its conversion to glutamate promotes cellular metabolism in human lymphoma cells and prostate cancer cells [174], also restraining the antitumor effects of macrophages [184]; supports epigenetic modifications in cellular FLICE-like inhibitory protein (cFLIP) and resistance of pancreatic tumor cells to apoptosis [262]; glutamine-derived aspartate supports EIF5a hypusination and HIF1α translation, promoting metabolic changes, i.e., enhanced glycolysis, in tumor-associated macrophages, which is correlated to advanced tumors [180]; promotes MDSCs recruitment and suppressive functions [181]; polarizes macrophages to M2 phenotype [183]; tumor cells-derived glutamate promotes aspartate synthesis by cancer associated fibroblasts, supporting the biosynthesis of nucleotides, and fueling tumor cells proliferation [185]; promotes the generation of suppressive memory B cells in tertiary lymphoid organs associated with tumors [176]; glutamine synthesis from glutamate, restrains both M1 macrophage differentiation and antitumor immunity [5, 182]; promotes the synthesis of GABA that stimulates the β-catenin pathway in tumor cells, fostering cellular proliferation and impairing CD8 + T cells antitumor activity [188]; its consumption by clear cell renal cell carcinoma in vitro leads to IL-23 secretion by macrophages and Tregs recruitment [189].	It is critical for NK cells [192] and Teffs responses, especially Th17 cells and CD8 + T cells, while its absence is associated with Tregs development [192–194]; promotes lactate generation and antitumor (mouse melanoma model) immune responses by macrophages [195]; its depletion leads to G-CSF and GM-CSF expression and subsequent MDSCs development [196]; as a source of glutathione, glutamine promotes resistance against immune suppression mediated by MDSCs-derived methylglyoxal [197].	Specific delivery of glutaminase inhibitors for TAMs or tumor cells can restrain glutamate-mediated inhibition of antitumor immunity and tumor cells proliferation, respectively. Glutamine supplementation directed to CD8 + T cells and NK cell can promote antitumor immunity. Antagonism of CD47 can impair glutamine-mediated tumor immune escape.

### Aspartate and Asparagine

Aspartate is a non-essential amino acid involved in distinct metabolic pathways (e.g., the urea cycle and the citric acid cycle) that has been recently associated with a tumorigenic role. In this context, increased aspartate levels in the lungs contribute to the establishment of cancer cells and lung metastases. Aspartate promotes collagen synthesis by cancer cells through its receptor NMDAR (same for glutamate, being a molecular target). Thus, aspartate leads to Eif5a hypusination and a translational program in cancer cells that supports TGFβ signaling, subsequent synthesis of collagen, and formation of an ECM that supports the establishment of metastasizing tumor cells [213]. In addition, N-acetyl-aspartate (NAA), generated by the activity of the enzyme N-acetyltransferase 8-like, restrains the cytotoxic activity of both CD8 + T cells and NK cells, contributing to the suppression of antitumor immunity against brain tumors. NAA exerts its function in a histone acetyltransferase P300/CBP-associated factor (PCAF)-dependent manner, leading to lamin A acetylation, impairing its interaction with Sun2 (Sad1 and Unc84 domain-containing protein 2) and subsequent lytic granules, from CD8 + T cells and NK cells, polarization and release [214]. These studies indicate that aspartate depletion might promote antitumor immunity and destabilize the tumor ECM. Furthermore, Elevated aspartate levels drive tumor proliferation by facilitating the synthesis of both nucleotides and asparagine [215, 216] (Table 9).

**Table 9:** The dual role of aspartate and asparagine in tumor biology (and immune responses)

Amino acids	Protumor effects	Antitumor effects	Therapeutic opportunities
**Aspartate**	Promotes collagen synthesis by cancer cells, contributing to metastases [213]; NAA inhibits CD8 + T cells and NK cells’ antitumor effects [214]; supports nucleotides and asparagine biosynthesis [215].	Promotes inflammasome activation and inflammatory responses [230–232].	Inhibition of aspartate and asparagine metabolism (e.g., enzymes involved in nucleotide and collagen synthesis) in tumor cells can have important antitumor effects. Asparagine and aspartate supplementation can enhance the antitumor effects of inflammasome activators [263].
**Asparagine**	Fuels cancer growth by anabolic reactions [217]; contributes to epithelial-mesenchymal transition and tumor metastases [5, 37]; its depletion promotes CD8 + T cells’ fitness [215, 221].	Promotes inflammasome activation and inflammatory responses [163]; optimizes TCR signaling and antitumor immunity of CD8 + T cells [233].	

The enzyme asparagine synthetase catalyzes the conversion of aspartate and glutamine to asparagine and glutamic acid. Meanwhile, cellular asparagine orchestrates anabolic metabolism and fuels cancer cell growth by regulating the uptake of other amino acids (including serine, arginine, and histidine). This regulation, in turn, coordinates mTOR activity, protein synthesis, serine metabolism, and nucleotide synthesis [217], critical to support cellular proliferation. Thus, asparagine supplementation limits autophagy and tumor adaptation, but also inhibits (through mTOR) AMPK-mediated p53 activation, contributing to breast tumor cells’ survival [5, 218]. In addition, asparagine enhances glutamine biosynthesis and epithelial-mesenchymal transition, contributing to breast tumor cells’ metastasis, especially in the absence of extracellular glutamine [5, 37]. Furthermore, asparagine can contribute to leukemic tumor cells metastasis (mouse model) through the enhancement of c-Myc expression [219]. Asparagine deprivation promotes ROS-dependent ATF4 expression in CD8 + T cells, leading to their functional improvement and antitumor immune responses in a mouse melanoma model [220, 221]. At last, asparagine can destabilize RIG-I, a cytosolic PRR involved in IFN-I release, after the recognition of exogenous or mitochondrial nucleic acids (RNAs), in macrophages. Therefore, asparagine impairs IFN-I release by murine bladder tumor cells, restraining antitumor immunity mediated by recruited CD8 + T cells [222].

In conjunction, these studies underscore that the inhibition of aspartate and asparagine metabolism, or their deprivation, might be a compelling therapeutic strategy against some tumors. In this sense, asparaginase, the enzyme involved in asparagine degradation, has been administered for the treatment of acute lymphoblastic leukemia, also being a compelling target for the treatment of mouse models of breast, lung, and colorectal cancers [223–225]. Accordingly, inhibition of the main enzyme (asparagine synthetase- ASNS) involved in asparagine biosynthesis can also restrain tumor cells development/proliferation by multiple mechanisms, including (i) weakening Nucks1 effects in the proliferation of osteosarcoma cells; (ii) increasing cell autophagy and inhibiting the mitogenic mitogen activated protein kinases (MAPKs) (JNK/ SAPK) pathway [226]; (iii) coordinating the protein and nucleotide synthesis (as an amino acid exchanger, for the import of serine) of murine breast tumor cells, lung tumor cells, hepatocellular carcinoma cells and liposarcoma, promoting the resistance to glutamine deprivation (in breast tumor cells and liposarcoma) and mTOR activation [227–229]. In the same direction, overexpression of ASNS seems to protect pancreatic tumor cells from apoptosis after cisplatin treatment and glucose restriction [226], and contribute to immune escape of murine bladder tumor cells [222]. ASSN also promotes hepatocellular carcinoma proliferation after activation of mTOR and the stabilization of the β-catenin/Wnt pathway [228].

Importantly, asparagine and aspartate can also promote the inflammatory effects of macrophages, after inflammasome activation, which might lead to antitumor immunity, depending on the circumstances [230–232]. Furthermore, in contrast to Chang et al. [220] and Gnaprakasam et al. [221], Wu et al. [233] described that asparagine can support the signaling downstream TCRs and antitumor immunity mediated by CD8 + T cells [233]. As discussed previously, fine-tuning TCR activation is critical for optimal activity of T cells, preventing anergy or exhaustion. These paradoxical effects might be at the central stage regarding the role of asparagine in T cells biology.

### Taurine

Taurine is a non-protein essential amino acid distributed in many tissues of animals, including the brain, heart, and other muscles. Several studies describe the effects of taurine on tumor and immune cells biology. In this sense, taurine is an important carbon and nitrogen source for certain tumor cells that express high levels of Slc6A6 (an interesting molecular target), the major transporter of taurine, and also for CD8 + T cells, being involved in T cells proliferation and antitumor immunity against melanoma (mouse model) [234]. In this sense, taurine consumption (the lack of taurine) by tumor cells leads to increased endoplasmic reticulum (ER) stress in infiltrated CD8 + T cells, subsequent expression of checkpoint receptors, and exhaustion [231], restraining antitumor (gastric cancer cells) immunity. Taurine also inhibits breast cancer cells’ invasiveness in vitro and in vivo in mouse models [235, 236], through the differentiation of inflammatory antitumor macrophages (M1). This probably explains why the use of morphine, which restrains taurine biosynthesis, can be associated with enhanced metastasis in individuals affected by breast cancer [237]. In these contexts, taurine supplementation might support antitumor CD8 + T cells and M1 macrophages. On the other side, taurine can support glycolysis and leukemogenesis, exerting protumor effects in hematopoietic tumors [238]. In general, as discussed above, taurine possesses a dual role, depending on the TME (including infiltrated immune cells) and the type of tumor cells (Table 10).

**Table 10:** The dual role of taurine in tumor biology (and immune responses)

Amino acid	Protumor effects	Antitumor effects	Therapeutic opportunities
**Taurine**	Supports tumor (Leukemic) cells’ metabolism [238].	Polarizes macrophages to M1 phenotype, inhibiting invasiveness [236, 237]; supports the metabolism (Oxphos) of antitumor CD8 + T cells, impairing exhaustion [234, 235].	Taurine supplementation can enhance macrophages and T cells-mediated antitumor immunity.

### Ammonia and other Nitro Derivatives of Amino Acids Degradation

The degradation of amino acids can lead to the generation of amine-containing compounds, like ammonia, and subsequently urea, that must be eliminated by the kidneys. Interestingly, ammonia detoxification by CD8 + T cells promotes their stemness, which is associated with antitumor immunity in a mouse melanoma model. The stemness of CD8 + T cells is mediated by the carbamoyl phosphate metabolic pathway, forming arginine in the cytosol [239]. As anticipated, arginine can be converted to nitric oxide by NOS (from the citrulline cycle) or directed to the mitochondria, where it is catabolized by arginase 2 to urea and ornithine (urea cycle). Both cycles share metabolic intermediates, like citrulline, arginosuccinate, and arginine, and are critical for ammonia detoxification, cellular survival, and stemness in CD8 + T cells [173]. Interestingly, the inability of CD8 + T cells to deal with excessive intracellular ammonia, originating from glutaminolysis, leads to mitochondrial and lysosomal damage and cell death. In this context, inhibition of glutaminolysis improves antitumor immunity after adoptive T cell transfer in a mouse model of melanoma [240].

Cadaverine, a short aliphatic diamine, is produced by microbiota after lysine degradation. Recently, cadaverine was shown to inhibit the endothelial-to-mesenchymal transition, invasion, and motility of breast cancer cells. This was mediated by the recognition of cadaverine by Trace amino acid receptors (TAAR1, TAAR8, and TAAR9) [241]. Cadaverine has also been implicated in transient receptor potential cation channel subfamily V member 1 (TRPV1) receptor activation in neurons, leading to itch [242]. As an agonist of TRPV1, cadaverine can possibly affect tumor cells distinctively, depending on their type [243]. In this sense, in rat models of lung cancer (squamous cell carcinoma of the lung), high plasma levels of cadaverine were associated with disease progression [244], a finding that might indicate its roles not only as a biomarker but also as a causative agent for this specific tumor. The role of cadaverine in the regulation of immune responses seems to be dependent on its concentration, at least in macrophages. Therefore, at high levels, cadaverine promotes histamine H4 receptor (H4R) signaling and a subsequent increase in the glycolytic pathway, which supports macrophage proinflammatory effects. On the other hand, at low levels, cadaverine is imported to the cytoplasm of macrophages by L-lysine transporters, inducing a modest increase in glycolysis that supports Oxphos and itaconate generation. Itaconate promotes Nuclear Factor Erythroid 2-related Factor 2 (Nrf2) activation and, subsequently, an anti-inflammatory phenotype in macrophages [245]. As proinflammatory macrophages can be associated with tissue damage, including tumors, it is expected that at high levels, cadaverine might also promote antitumor immunity in the context of tumor-associated macrophages.

### Peptides

#### Carnosine

Carnosine is a naturally occurring dipeptide. It is found in muscle cells and is considered an anti-glycating amino acid composed of beta-alanine and histidine. The effects of carnosine in tumor biology, like many other metabolites, seem dichotomous. Carnosine synthesis by an unrecognized carnosine synthase (CARNS2) is critical for the adaptation of tumor cells to hypoxia, maintaining pH homeostasis and lysosome activity. Through this, carnosine promotes tumor-mediated immune evasion, facilitating nuclear transcription factor X-box binding 1 (NFX1)-lysosomal degradation and subsequent galectin-9 expression [246] in a mouse model of hepatocellular carcinoma. However, carnosine has also been described to promote phagocytosis-related resolution [247], which might contribute to antitumor immunity after the phagocytosis of tumor cells (detached from the tumor mass). Furthermore, carnosine possesses anti-proliferative effects in vitro for different cancer cell lines, i.e., breast, ovarian, colon, and leukemic cancer cells, and promotes chemokine expression by them [248], which might be associated with immune cells recruitment. As discussed throughout this text, the effects of immune cells’ recruitment can be dual, depending on their type, associated with suppressive or proinflammatory-antitumor effects.

#### Substance P

Substance P is a neuropeptide that has been described to possess diverse immunomodulatory roles, promoting phagocytosis and NK cells cytotoxicity [249], probably contributing to antitumor immunity. Recently, the effects of substance P in the induction of breast tumors metastasis have been described. Breast tumors promote substance P secretion by adjacent sensory neurons, leading to cell death of a small proportion of the tumor cells, subsequent single-stranded RNA release, TLR7-mediated signaling, and metastasis of the surviving tumor cells [250]. Therefore, although substance P is expected to induce antitumor immune responses, it can also promote tumor metastasis, at least in mouse models of breast tumors.

## Conclusions

As discussed above, the role of amino acids in supporting or inhibiting tumor biology and antitumor immune responses can be variable, depending on the tumor type, its mutations, infiltrated immune cells, the presence of other molecules, the biochemical conditions in the TME (oxygen levels, etc), and the metabolism of the cells from the TME, influencing, for example, how they use the amino acids, as a carbon source or as a substrate for enzymes or PTMs. Therefore, despite being compelling candidates for antitumor therapies, dietary amino acids supplementation or the inhibition of transporters/receptors or enzymes involved in the metabolism of amino acids must be appropriately and specifically selected according to the tumor type. In animal models and in the clinical setting, distinct pharmaceuticals have been described to modulate the function of transporters or enzymes involved in the metabolic dynamics of amino acids (Table 11). For instance, tamoxifen is known to inhibit one of the transporters of glutamine [251], and epacadostat can inhibit the degradation of tryptophan by IDO1. In this sense, adjunctive therapies for specific tumors using these modulators can further improve immunotherapies. To avoid collateral effects, more specific modulators of amino acids metabolism can be developed, through the use of monoclonal antibodies, mimetic peptides that activate receptors, proteolysis-targeting chimera degraders, and/or antisense nucleotides targeting genes (receptors, transporters, or enzymes) [252–255]. These selective therapies can also be targeted to act specifically in immune cells within the TME [176], avoiding their antagonistic and undesired effects when affecting other cells, like tumor cells. The use of programs, like Kaplan-Meier Plotter, that correlate the expression levels of genes involved in the metabolism of amino acids with the prognosis of different tumors might indicate the candidate tumors for these adjunctive therapies, especially when more than one gene is associated with the same outcome (good or bad prognosis), similar to a “gene enrichment score”, but for prognosis. For instance, the Kaplan-Meier Plotter analysis of human kidney renal papillary cell carcinoma (Parameters: Start Km Plotter for pan-cancer RNAseq; Overall survival; Trichotomization: Q1vsQ4) reveals that a high expression of four genes (TDO2, IDO1, IDO2, and Slc7a11) involved in kynurenine synthesis, from tryptophan, and transport is associated with a worse prognosis [256]. Thus, based on the here discussed mechanistic insights from animal studies, it is possible that this association is not fortuitous, and the inhibition of IDO1 might be a compelling adjuvant therapeutic strategy in this case. Finally, as tumor cells and immune cells are both affected by these modulators, cell-type-specific delivery of them with nanostructured lipid carriers or modular envelope design [257, 258] might yield better therapeutic results, allowing, for example, the expected inhibitory function upon metabolism in tumor cells, without affecting immune cells-mediated antitumor immunity.

**Table 11:** Therapeutic interventions targeting amino acids metabolism

Molecular Target	Pharmaceuticals	Tumor type
Enzyme: IDO1, involved in tryptophan degradation and kynurenine generation	Inhibitors: 1-methyl-D-tryptophan* (there are controversies related to its inhibitory activity [264, 265]); Epacadostat* (*on clinical trials/ NCT00567931; NCT03361865); Navoximod; HTI-1090; PEG-kynyreninase	Cancers in which the expression of IDO1 and other enzymes and transporters of tryptophan metabolism is associated with poor survival.
Membrane transporter: Slc7A11, Slc7a5 (also involved in BCAAs transport) involved in kynurenine transport	Inhibitors: Sulfasalazine; Vitamin D: Slc7a11 IGN523: Antagonistic anti-Slc7a5	Cancers in which the expression of Ahr is associated with poor survival.
Enzyme: Arginase- degradation of arginine Arginine Deiminase- arginine depletion	Arginase inhibitors: S-(2-boronoethyl)-L-cysteine (BEC); Ergotamine; Fludarabine, CB-1158* (*NCT02903914) Administration of PEG Arginine Deiminase (ADI-PEG20)	Cancers in which the expression of arginase and arginine transporters is associated with poor survival. Interestingly, BEC does not inhibit iNOS, allowing for the antitumor effects of reactive nitrogen species.
Arginine derived Nitric oxide	Promoter of NO: Tetrahydrobiopterin	Cancers in which the expression of NOS (but not arginases) and arginine transporters is associated with good prognosis.
Membrane transporter: Slc1a5, glutamine transporter	Inhibitors: Tamoxifen; Raloxifene.	Cancers in which the expression of Slc1a5 and enzymes from the metabolism of glutamine is associated with poor prognosis; enhanced expression of glutamine transporters in T cells for outcompeting tumor cells for glutamine. Approved against breast cancer [10].
Enzyme: Glutamine metabolism	Inhibitor of glutaminase: CB-839 (or Telaglenastat) Glutamine antagonist: DRP-104 (or Sirpiglenastat)	Cancers in which the expression of glutaminase and glutamine transporters is associated with poor prognosis; identifying the major cells expressing glutaminase by single-cell transcriptomics would indicate if the use of an inhibitor would be a compelling treatment strategy, e.g., if tumor cells are the major ones expressing glutaminase.
Enzyme: Asparaginase (asparagine degradation)	Administration of asparaginase: Asparlas Dietary asparagine restriction	Cancers in which the expression of asparaginase and other enzymes/transporters from asparagine metabolism is associated with a good prognosis. Asparlas is approved for use in Acute lymphoblastic leukemia [10].
Enzyme: Cysteinase (cysteine degradation)	Administration of cysteinase/ dietary cysteine restriction	Cancers in which the expression of cysteinase and enzymes/transporters involved in cysteine metabolism is associated with a good prognosis.
Enzyme: AOC3 involved in the generation of methylglyoxal	Inhibitor: Hydralazine and isoniazid	Cancers in which the expression of AOC3 and enzymes involved in glutamine degradation is associated with poor prognosis.
Enzyme: Methionine metabolism; fatty acid oxidation (FAO)	Inhibitor: Ranolazine	Cancers in which the expression of enzymes involved in fatty acid oxidation and/or methionine metabolism is associated with poor prognosis.
Enzyme: Involved in one carbon metabolism	SAM supplementation; PRMT inhibitors (PRT811, JNJ- 64,619,178, GSK3326595, and PF-06939999)	Cancers in which the expression of enzymes from one-carbon metabolism or transporters of amino acids that support the folate cycle is associated with a better prognosis.
Enzyme: Anabolism of amino acids	Amino acid supplementation	Cancers in which the enzymes involved in amino acid catabolism are associated with a poor prognosis; or in which enzymes involved in amino acids biosynthesis are associated with a good prognosis.

## Data Availability

No datasets were generated or analysed during the current study.
